# Nanopatterning of steel by one-step anodization for anti-adhesion of bacteria

**DOI:** 10.1038/s41598-017-05626-0

**Published:** 2017-07-13

**Authors:** Shiqiang Chen, Yuan Li, Y. Frank Cheng

**Affiliations:** Department of Mechanical & Manufacturing Engineering, University of Calgary, Calgary, Alberta T2N 1N4 Canada

## Abstract

Surface nanopatterning of metals has been an effective technique for improved performance and functionalization. However, it is of great challenge to fabricate nanostructure on carbon steels despite their extensive use and urgent needs to maintain the performance reliability and durability. Here, we report a one-step anodization technique to nanopattern a carbon steel in 50 wt.% NaOH solution for highly effective anti-adhesion by sulphate reducing bacteria (SRB), i.e., *Desulfovibrio desulfuricans subsp. desulfuricans* (Beijerinck) Kluyver and van Niel. We characterize the morphology, structure, composition, and surface roughness of the nanostructured film formed on the steel as a function of anodizing potential. We quantify the surface hydrophobicity by contact angle measurements, and the SRB adhesion by fluorescent analysis. The optimal anodization potential of 2.0 V is determined for the best performance of anti-adhesion of SRB to the steel, resulting in a 23.5 times of reduction of SRB adhesion compared to bare steel. We discuss the mechanisms for the film formation on the steel during anodization, and the high-performance anti-adhesion of bacteria to nanopatterned steels. Our technique is simple, cost-effective and environment-friendly, providing a promising alternative for industry-scale surface nanopatterning of carbon steels for effective controlling of bacterial adhesion.

## Introduction

Steels have been the most widely used engineering materials in current civilization due to their availability, economic benefits, and unreplaceable mechanical property^1^. However, the steels usually suffer from degradation by various mechanisms when exposed to aqueous environments. Particularly, microbiologically influenced corrosion (MIC) and biofouling are two primary mechanisms resulting in detrimental effects on steel structures and facilities in a wide variety of industrial sectors, including oil/gas production and transportation, ships, aquaculture systems, heat exchangers, etc^2–4^. Statistics showed that MIC is responsible for nearly 50% of corrosion scenarios in oil/gas pipelines^5^. A recent analysis of the economic impact of biofouling for entire U.S. navy fleet estimated that the approximate cost is between USD $180 and 260 million per year^6^. It is generally accepted that bacterial attachment is the first step for the formation and growth of biofilm, resulting in MIC and biofouling of the steels^7, 8^.

Of various microorganisms that can adhere to metals (steels), SRB, one type of anaerobic bacteria using sulphate as a terminal electron acceptor to degrade organic compounds, are widely spread in environments and easily form a biofilm on the steel surface^4, 9^. Corrosion of the steels occur under the biofilm through several mechanisms, such as cathodic depolarization theory, concentration and galvanic cell formation, and direct electron transfer^10^. It was estimated^5^ that corrosion loss induced by SRB accounts for over 50% of all MIC. In addition to corrosion, the presence of SRB is responsible for biofouling in varied applications, such as cooling water pipes, heat exchange tubes, pipelines, etc., resulting in significant economic loss^11–13^. Development of surface techniques for anti-adhesion of SRB to the steel provides a potential method for alleviation of MIC and biofouling.

Nanoscale surface topography enables controlling or even elimination of bacterial adhesion to metals by affecting the bacteria-substrate interaction^3, 14^. This provides an environment-friendly alternative that avoids introduction of chemicals such as biocides or inhibitors to the environment to potentially result in toxicity and low durability of the system. Electrochemical approaches, such as anodization and electrodeposition, have been demonstrated as efficient and convenient methods to generate nanostructures on metals^15–18^. They enable coherent growth of surface films on the substrate, and ensure well binding between the layers. Primarily, electrochemical fabrication of nanostructure are conducted on metals such as aluminum, titanium and their alloys, as well as stainless steels^17, 18^, which are easily passivated to form a uniform passive film on the metal surface. Some work used fluorinated chemicals, which are environmentally hazard and difficult to treat, in order to achieve the nono-patterning^16^. To date, nanopatterning by “green” electrochemical methods on carbon steels, the most commonly used engineering materials, has been rarely reported. This is attributed to the facts that the carbon steels are usually electrochemically active in most environments. Moreover, they are chemically and structurally non-uniform at the nanometer scale. The anisotropy of the steels affects the growth of the surface film, usually resulting in an inhomogeneous film.

In this work, we use a one-step anodization technique to develop nanostructured films on the surface of an X100 carbon steel in 50 wt% NaOH solution. The morphology, structure and composition of the film are characterized by scanning electron microscopy (SEM), atomic force microscopy (AFM) and Raman spectroscopy. We measure the contact angle of the nanopatterned surface of the steel to determine its hydrophobicity. Moreover, we quantify the SRB adhesion to the steel as a function of anodizing potential by fluorescent analysis. The mechanistic aspects for the formation of nano-film on the steel during anodization, and the effective anti-adhesion to SRB on the nanopatterned steel are discussed. We demonstrate that the technique reported in this work provides a promising alternative for surface nanopatterning of carbon steels, effectively controlling bacterial adhesion and prevention of MIC and biofouling to maintain the integrity of facilities.

## Results

### Characteristics of the nanostructured film formed on the anodized steel

**Figure 1:**
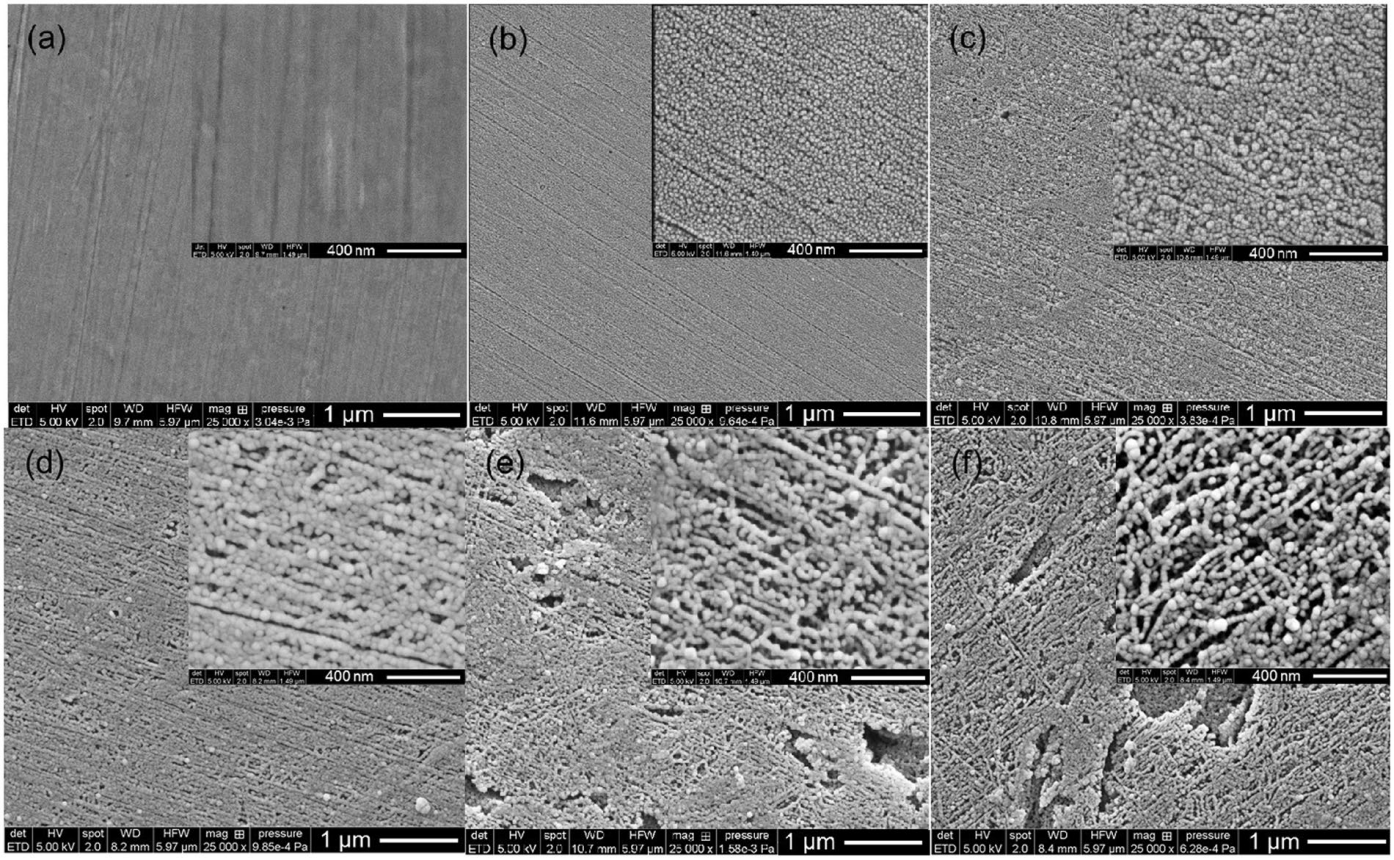
SEM views of the morphology of the nanostructured films. (**a**) Bare steel, **(b**–**f)** Films formed at the anodizing potentials of 1.0 V, 1.5 V, 2.0 V, 3.0 V and 4.0 V, respectively. For each anodizing condition, photos taken at magnifications of 25,000 times and 100,000 times are given.

**Figure 2:**
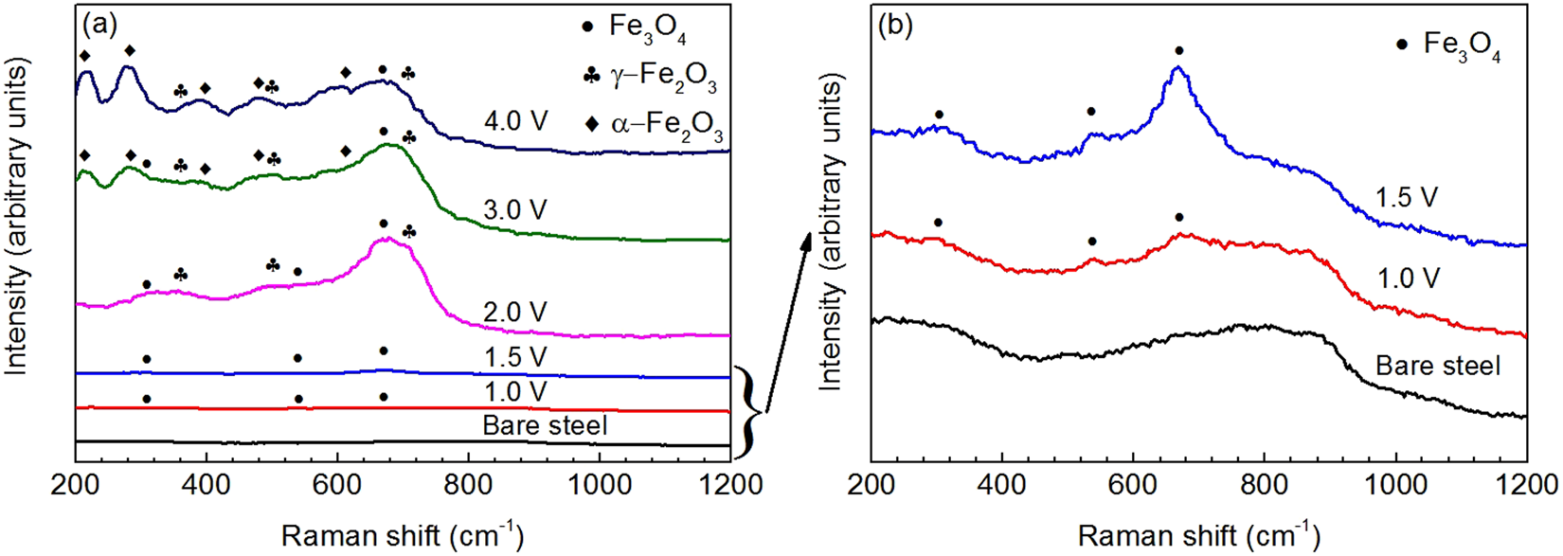
Compositional characterization of the films by Raman spectroscopy. (**a**) Bare steel and films formed at various anodizing potentials. (**b**) Enlarged spectra measured on the bare steel and the steels anodizing at 1.0 V and 1.5 V.

We prepare nanostructured films on the surface of X100 carbon steel in 50 wt% NaOH solution by one-step anodization at various potentials. Figure 1 shows the SEM view of the morphology of the films formed at various anodizing potentials at 30 °C for 10 min, where photos taken at two magnifications (i.e., 25,000 and 100,000 times) are given for each anodizing condition. For compositional characterization on the films, the Raman spectroscopy is used and the results are shown in Fig. 2. It is noted that the SEM photos represent the ones with the best quality of numerous images taken by the equipment. The anodized films do not have a proper conductivity. Moreover, the presence of magnetic components, such as Fe_3_O_4_, in the film makes it very difficult to obtain a better picture at the high magnitude of 100,000 times. Obviously, the morphological feature and composition of the nanostructured films depend heavily on the anodizing potential. For bare steel, the surface is flat and smooth (Fig. 1a). There is no iron oxide formed, where the broad peak from 700 to 900 cm^−1^ is from the environment^19^, as seen in Fig. 2b. When the anodizing potential is 1.0 V, a uniform, compact film containing fine nanoparticles with the average diameter of about 37 nm is formed on the steel surface (Fig. 1b). The film is composed of magnetite (Fe_3_O_4_), as indicated by a broad band peak around 670 cm^−1^ and another two weak broad peaks around 538 cm^−1^ and 306 cm^−1^ in the Raman spectrum (Fig. 2b)^20^. The open circuit potential (OCP) of the steel anodized at 1.0 V in phosphate buffered solution (PBS) drops rapidly from the initial −74 mV vs. saturated calomel electrode (SCE) to the steady value of −712 mV vs. SCE after 18 h of immersion, as shown in Supplementary Fig. 3. This indicates that the film formed at the anodizing potential of 1.0 V is active and cannot maintain stable in the solution.

When the steel is anodized at 1.5 V, a uniform film containing larger nanoparticles is formed, as seen in Fig. 1c. The Raman result shows that the main component of the film is also Fe_3_O_4_, but the intensity of the peaks becomes stronger (Fig. 2b), indicating that the film contains more Fe_3_O_4_ at the anodizing potential of 1.5 V than at 1.0 V. The OCP measurement on the steel anodizing at 1.5 V gives the steady state value of −136 mV vs. SCE (Supplementary Fig. 3), which is much less negative than the steady OCP of −712 mV vs. SCE when the steel is anodized at 1.0 V, indicating the increased stability of the film in the solution. At the anodizing potential of 2.0 V, the size of nanoparticles and the feature of the film are different from that anodized at 1.5 V. The nanoparticles with an average diameter of around 60 nm distributes uniformly on the film. Some irregular nanopores with the diameter less than 150 nm and nanocracks with a width about 15 nm can be observed in Fig. 1d. The Raman spectrum indicates that, in addition to Fe_3_O_4_, maghemite (γ-Fe_2_O_3_) is also formed on the steel that is anodized at 2.0 V, as indicated by the new peaks at 360 cm^−1^, 500 cm^−1^ and 704 cm^−1^ (Fig. 2a)^20, 21^. The intensity of the Fe_3_O_4_ and γ-Fe_2_O_3_ peaks are stronger, which means that there are more iron oxides produced in the film when formed at 2.0 V. The steady-state OCP of the steel anodizing at 2.0 V is −121 mV vs. SCE, which becomes further less negative, demonstrating the improved stability of the film anodizing at 2.0 V, as seen in Supplementary Fig. 3.

When the anodizing potential is increased to 3.0 V and 4.0 V, while the size of nanoparticles remains unchanged compared to that obtained at 2.0 V, the nanopores become bigger. The integrity of the film is worse along with the presence of broken areas in the films, as seen in Fig. 1e and f. In addition to Fe_3_O_4_ and γ-Fe_2_O_3_, hematite (α-Fe_2_O_3_) is formed on the film, which is indicative of the new peaks around 220 cm^−1^, 282 cm^−1^, 397 cm^−1^, 487 cm^−1^ and 604 cm^−1^ in Fig. 2a ^20, 22^. The intensity of the peaks for α-Fe_2_O_3_ at 3.0 V is smaller than that at 4.0 V, showing that the amount of α-Fe_2_O_3_ in the film increases with the anodizing potential. The stability of the film increases with the anodizing potentials, as indicated by the more positive OCP in Supplementary Fig. 3. The OCP values of the filmed steel are −121 mV vs. SCE, −70 mV vs. SCE, and 90 mV vs. SCE at the polarizing potentials of 2.0 V, 3.0 V and 4.0 V, respectively. This behavior is related to the increase of the film thickness as the anodizing potential increases. As shown in Supplementary Fig. 4, the thickness of the film increases with the anodizing potential. For example, the thicknesses of the film at potentials of 1.0 V, 1.5 V, 2.0 V, 3.0 V and 4.0 V are about 1 µm, 2 µm, 9 µm, 11 µm and 15 µm, respectively.

**Figure 3:**
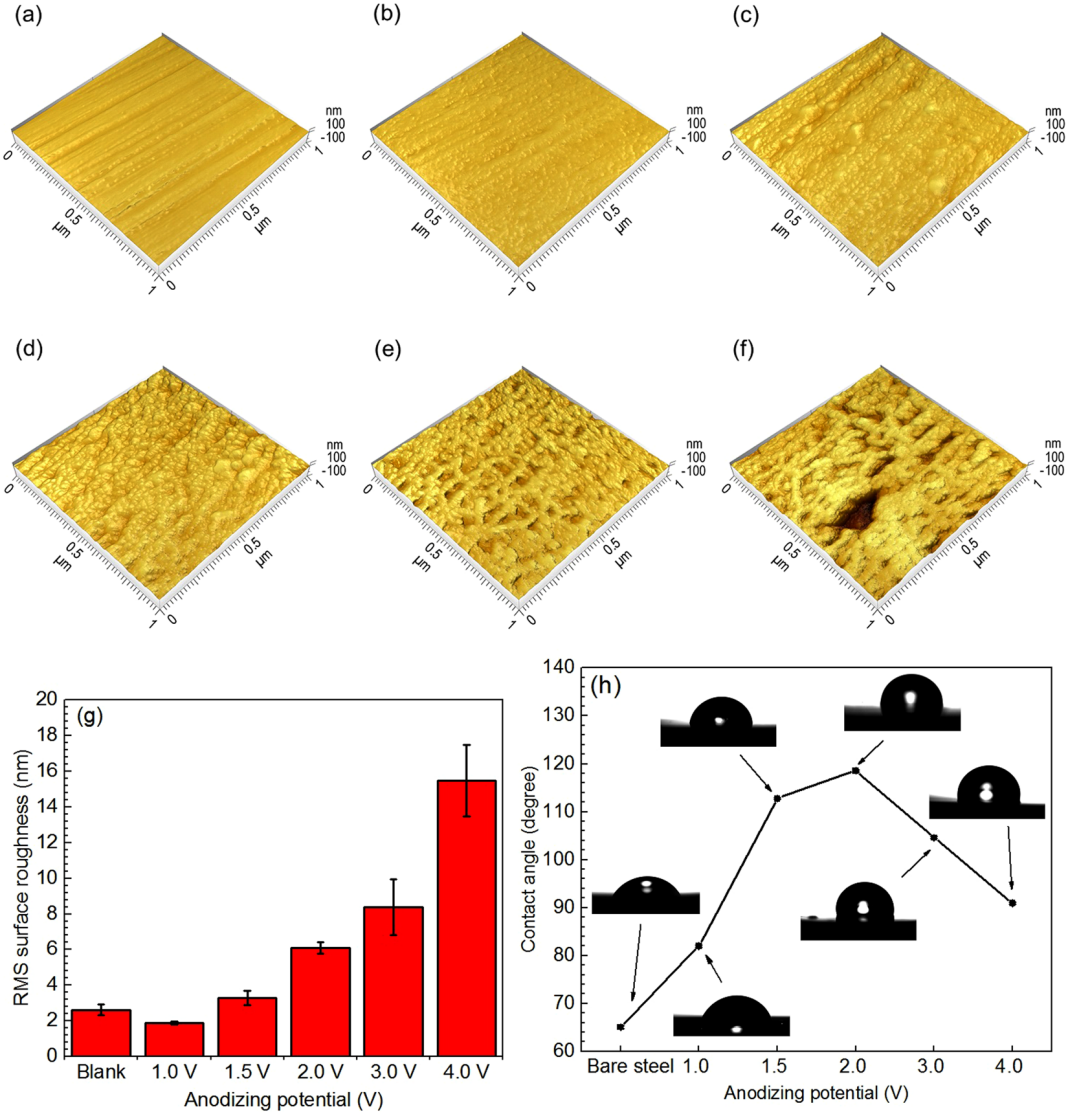
Surface topography, roughness and contact angle of the nanostructured films. **(a–f)** AFM images of bare steel and the steel anodizing at 1.0 V, 1.5 V, 2.0 V, 3.0 V and 4.0 V, respectively, in 50 wt% NaOH solution at 30 °C for 10 min. **(g)** Surface roughness of bare steel and the steel anodizing at various potentials. **(h)** The contact angles of bare steel and the steel anodizing at various potentials.

The AFM topographic images of the steel anodizing at various potentials are shown in Fig. 3a–f. The results are well consistent with the SEM views in Fig. 1. It is seen that, with the anodizing potential increasing from 1.0 V to 1.5 V and 2.0 V, the nanostructured film is compact and uniform, along with the increasing size of nanoparticles (Fig. 3b–d). When the potential is up to 3.0 V and 4.0 V, holes and broken areas are present on the film (Fig. 3e and f). The surface roughness of the anodized steels at various potentials is shown in Fig. 3g, where the root-mean-square (RMS) roughness is derived from the AFM topographic profile of the surface films. It is seen that the film formation reduces the surface roughness of the bare steel from 2.62 nm to 1.87 nm. This is attributed to the fact that the nanoscale film can eliminate topographic irregularities present on the steel substrate^23^. With the increases of the anodizing potential, the surface roughness increases, which is due to the growing nanoparticles and the presence of holes/broken areas on the film, especially at high anodizing potentials. The hydrophobicity of the nanostructured film is characterized by contact angle measurements, and the result is shown in Fig. 3h. The water contact angle of the bare steel is about 65.08°, indicating that the steel is hydrophilic. Upon anodization, the contact angle increases. At the anodizing potentials of 1.0 V, 1.5 V and 2.0 V, the contact angles are 81.98°, 112.90° and 118.53°, respectively. However, with the further increase of the potential to 3.0 V and 4.0 V, the contact angle decreases to 104.43° and 91.16°, respectively. Thus, the anodization treatment is able to improve the hydrophobicity of the steel. The maximum hydrophobicity is achieved when anodizing the steel at 2.0 V, as indicated by the largest contact angle.

### Anti-adhesion to SRB on the nanopatterned steel

**Figure 4:**
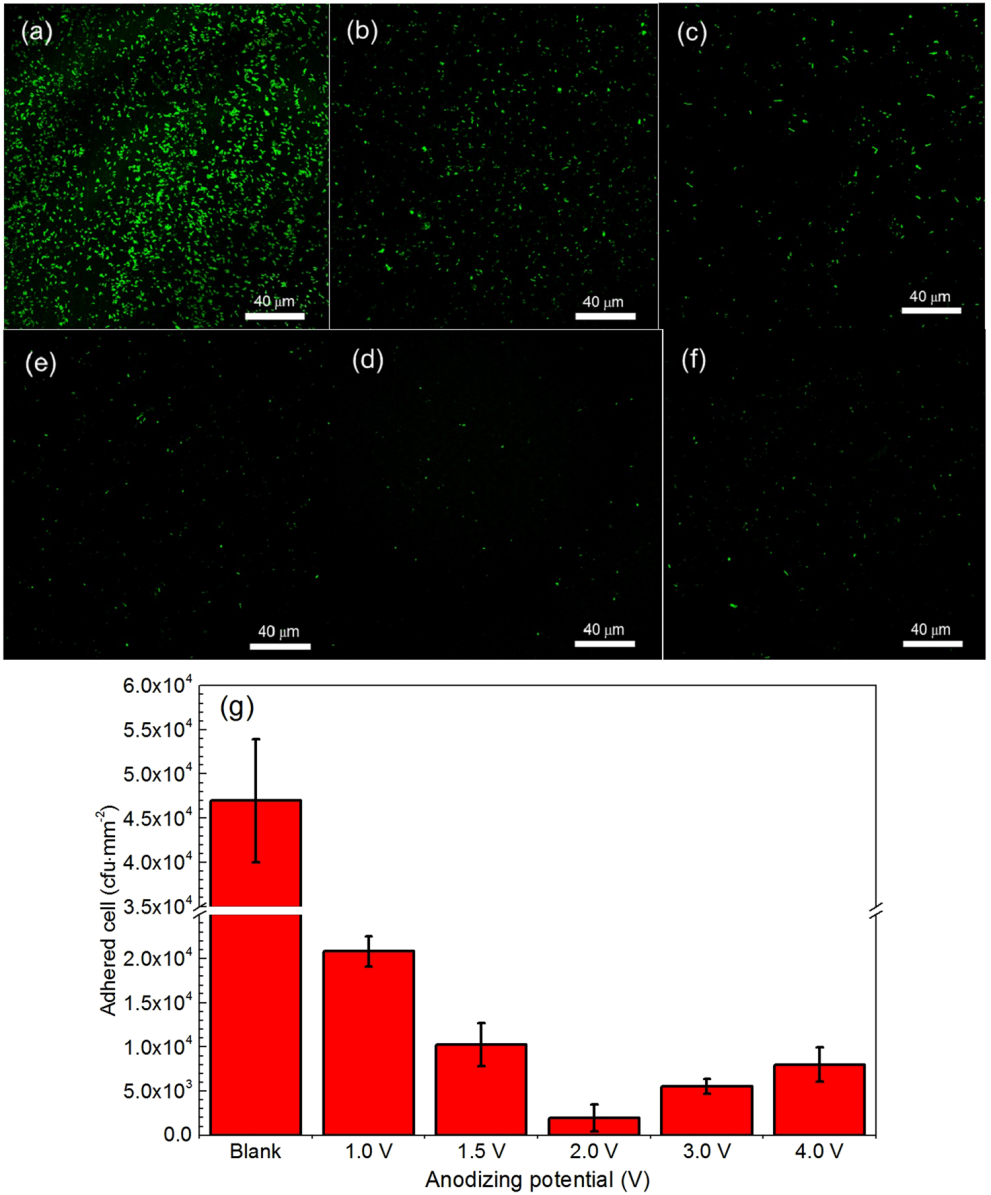
Fluorescent images of bare steel and the nanopatterned steels and the statistical quantity of adhered SRB cells. (**a–f**) The fluorescent images of bare steel and the anodized steels at 1.0 V, 1.5 V, 2.0 V, 3.0 V and 4.0 V, respectively, taken after 18 h of immersion in SRB-containing PSB solution. **(g)** Statistic results of the quantity of SRB cells adhered on the steels anodizing at various potentials.

To characterize the anti-adhesion to SRB by the nanopatterned steel, we conduct the fluorescent accounting of vital bacteria on the surface of anodized steel specimens. Figure 4 shows the fluorescent images of bare steel and the steel anodizing at various potentials after 18 h of immersion in SRB-containing PSB solution and the statistic results of the quantity of adhered SRB cells on the steel. Obviously, SRB cells adhere extensively on the bare steel surface (Fig. 4a), and the quantity of the adhered SRB cells is about 4.7 × 10^4^ cfu/mm^2^. In addition to SRB cells, there are abundant of extracellular polymeric substances (EPS) and corrosion products present on the steel surface as well, as seen in Supplementary Fig. 6a. Thus, the X100 carbon steel is vulnerable to SRB cell adhesion and corrosion. Upon anodization to form a layer of nanostructured film, the SRB adhesion is reduced remarkably. When the steel is anodized at 1.0 V and 1.5 V, the density of SRB cells adhered on the steel surface decreases obviously (Fig. 4b and c). From the statistic analysis, the density of adhered SRB cells on the steel anodized at 1.0 V and 1.5 V are 2.09 × 10^4^ and 1.0 × 10^4^ cfu/mm^2^, respectively. Moreover, the EPS and corrosion products decrease with the increased anodizing potential, as shown in Supplementary Fig. 6b and c. When the anodizing potential is increased to 2.0 V, there is a further decrease of the density of adhered SRB cells (Fig. 4g), and the amount is 2.0 × 10^3^ cfu/mm^2^ only. However, when the anodizing potential is up to 3.0 V and 4.0 V, the amount of SRB cells on the steel surface increases slightly (Fig. 4g), and the density of adhered SRB cells are 5.6 × 10^3^ and 8.1 × 10^3^ cfu/mm^2^, respectively. There is no EPS adhered on the steel anodized at 2.0 V, 3.0 V and 4.0 V (Supplementary Fig. 6d–f). Thus, an optimal anodizing potential exists, where the nanostructured film formed on the steel possesses the best performance against SRB adhesion. In this work, the optimal anodizing potential is 2.0 V for X100 steel. Compared to bare steel, there is a 23.5 times of reduction of the quantity of adhered SRB cells to the nanopatterned steel anodizing at 2.0 V.

## Discussion

### Formation of nanostructured films by anodization of carbon steel

**Figure 5:**
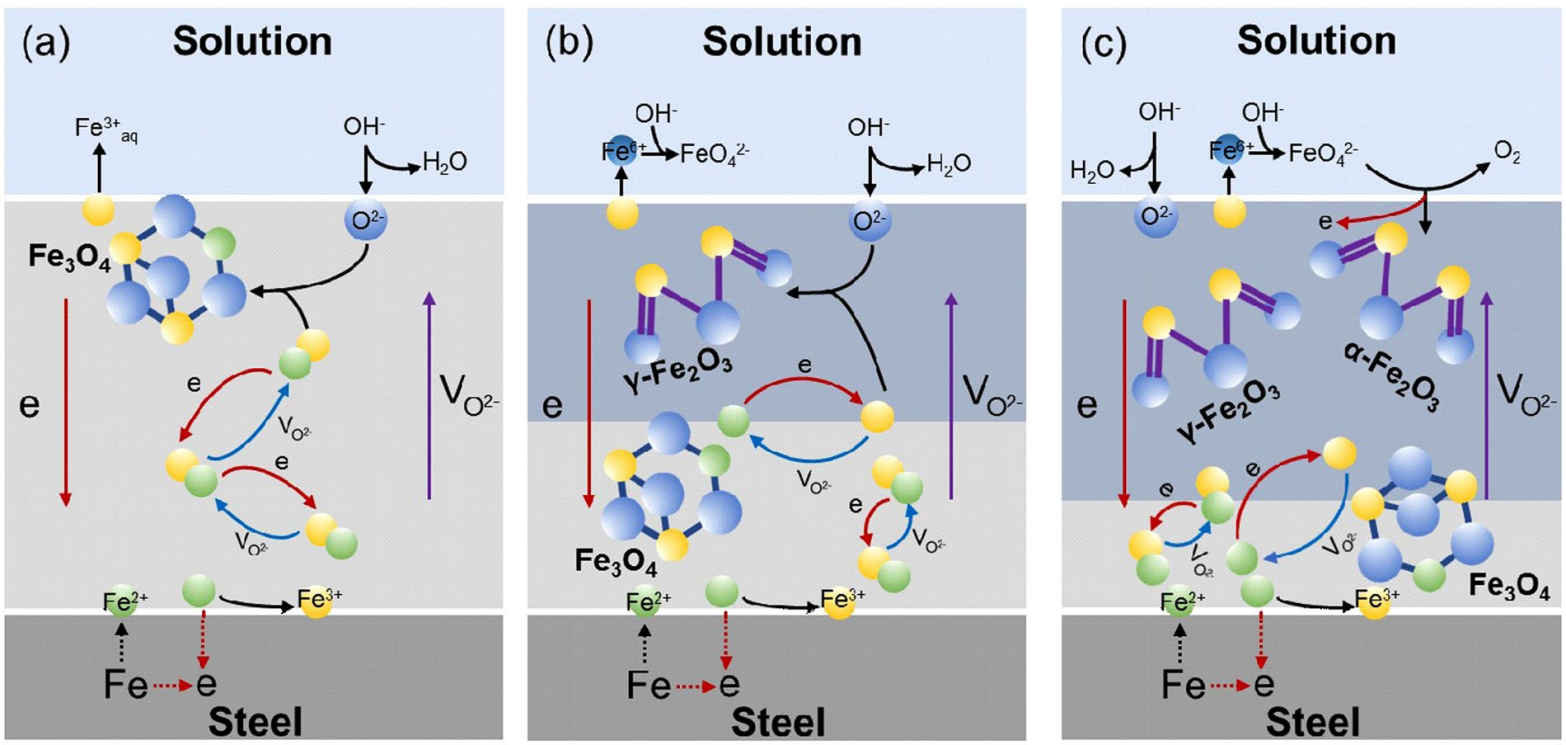
Mechanisms for the formation of nanostructured film during anodization of carbon steel. (**a**) Formation of Fe_3_O_4_ at 1.0 V and 1.5 V. (**b**) Formation of Fe_3_O_4_ and γ-Fe_2_O_3_ at 2.0 V. (**c**) Formation of Fe_3_O_4_, γ-Fe_2_O_3_ and α-Fe_2_O_3_ at the anodizing potentials of 3.0 V and 4.0 V. Yellow balls refer to Fe^3+^ ions, green balls for Fe^2+^ ions, blue balls for O^2−^ ions, and mazarine balls for Fe^6+^ ions.

The anodic reaction of carbon steels in an alkaline solutions is oxidation of iron to Fe^2+^, Fe^3+^ and Fe^6+^ along with the increasing potential, where Fe^6+^ is present in the form of FeO_4_^2−^ in alkaline solutions. Analysis of electrolyte after anodization at various potentials shows that FeO_4_^2−^ is not formed under low potentials (i.e., 1.0 V and 1.5 V), as shown in Supplementary Fig. 2a and b. When the anodizing potential exceeds 2.0 V, the FeO_4_^2−^ concentration increases with the potential (Supplementary Fig. 2c–e). This work finds that, at the potentials of 1.0 V and 1.5 V, the formed iron oxides are mainly Fe_3_O_4_. According to the proposed mechanism illustrated in Fig. 5a, the oxidation of Fe to Fe^2+^ and Fe^3+^ takes place on the steel surface by:

1 $${\rm{Fe}}\to {{\rm{Fe}}}^{2+}+{\rm{2e}}$$

2 $${{\rm{Fe}}}^{2+}\to {{\rm{Fe}}}^{3+}+{\rm{e}}$$

According to point defect model (PDM)^24, 25^, electrons and oxygen vacancies (V_O_^2−^) are generated by converting Fe^2+^ to Fe^3+^. V_O_^2−^ migrate towards the film/solution interface, where O^2−^ is injected into the outer layer, causing dissolution of Fe oxide to generate Fe^3+^. Anodization of the steel at 1.0 V and 1.5 V results in formation of iron oxide with an average valence equivalent to Fe_3_O_4_, and the film grows until the transport flux of charge species through the oxide becomes equivalent to the dissolution rate of Fe^2+^ to Fe^3+^ at the film/solution interface, as shown in Fig. 5a.

When the anodization potential is increased to 2.0 V, in addition to the formation and growth of Fe_3_O_4_, the conversion of Fe_3_O_4_ to γ-Fe_2_O_3_ becomes feasible since both oxides have a similar crystallographic structure^26^. It has been confirmed that oxide films can be composed of a Fe_3_O_4_ inner layer and a Fe_2_O_3_ outer layer^27^. As illustrated in Fig. 5b, after formation of Fe_3_O_4_, it is converted to γ-Fe_2_O_3_ through the migration of V_O_^2−^ towards the γ-Fe_2_O_3_/solution interface, followed by injection of O^2−^ into the oxide matrix. During this process, some Fe^3+^ loss electrons to form Fe^6+^, which reacts with OH^−^ to form FeO_4_^2−^:

3 $${{\rm{Fe}}}^{3+}+8{{\rm{OH}}}^{-}\to {{{\rm{FeO}}}_{4}}^{2-}+4{{\rm{H}}}_{2}{\rm{O}}+3{\rm{e}}$$

This is confirmed by the low concentration of FeO_4_^2−^ (0.023 mmol/L) measured in the electrolyte, as seen in Supplementary Fig. 2c. Thus, the surface film formed at 2.0 V is a mixed Fe_3_O_4_/γ-Fe_2_O_3_ oxide, with Fe_3_O_4_ in the inner region and γ-Fe_2_O_3_ in the outer region of the film.

When the anodizing potential increases to 3.0 V and 4.0 V, as shown in Fig. 5c, the ferric oxides loss electrons at the oxide/solution interface to produce FeO_4_^2−^, which reacts to form α-Fe_2_O_3_ and O_2_, as indicated by the presence of bubbles during anodization at 3.0 V and 4.0 V^28^:

4 $$4{{{\rm{FeO}}}_{4}}^{2-}\to 2{{\rm{Fe}}}_{2}{{\rm{O}}}_{3}+5{{\rm{O}}}_{2}+8{\rm{e}}$$

The α-Fe_2_O_3_ deposits on the steel surface, as shown by the high intensity of characteristic peaks of α-Fe_2_O_3_ in Fig. 2a. The increased surface roughness of the nanostructured film and the reduced electrochemical stability, compared to the film formed at the anodizing potential of 2.0 V, are associated with the dissolution of ferric oxides.

### Anti-adhesion to SRB on the nanopatterned steel

Bacterial adhesion and the biofilm formation are essential to MIC occurrence and biofouling of metals. The adhesion of a bacterial cell to the metal is governed by several factors, including the physicochemical properties of the bacteria and the metal, and the environmental conditions^29, 30^. In this work, the physicochemical property of the X100 carbon steel is the dominate factor for SRB adhesion under the given testing condition. Both SEM and OCP results show that the bare steel and the anodized steel at 1.0 V and 1.5 V are not sufficiently stable in SRB medium, as seen in Supplementary Fig. 3 and Supplementary Fig. 6a–c. The Fe^2+^ ions formed during steel corrosion can react with EPS^31^, which are produced through SRB metabolism and have a high complexation activity, facilitating the SRB adhering to the steel^32^. Thus, extensive SRB cells are observed on the surface of bare steel and the steel anodizing at 1.0 V and 1.5 V (Fig. 4).

The steel anodizing at 2.0 V, 3.0 V and 4.0 V is stable in the SRB-containing medium. Generally, the surface roughness and hydrophobicity are two important properties affecting the bacterial adhesion to metals^33^. Extensive studies have shown that bacterial attachment is directly related to the surface roughness at nanoscales^34, 35^. The steel anodizing at 2.0 V shows the best performance for anti-adhesion by SRB due to the smallest surface roughness compared to those anodizing at other potentials. As shown in Supplementary Fig. 6, the adhesion of SRB (i.e., gram-negative bacteria) on the steel specimens anodized at 2.0 V, 3.0 V and 4.0 V shows a similar characteristic, i.e., a hair-like nanofiber is present between the SRB cell and the film (see the insert figures). This is called pilus and fimbria, the most well-known proteinous adhesion for gram-negative bacteria. The adhesion of this protein on nanoscale surfaces depends closely on the surface roughness^36^. With an increase of the surface roughness, the fraction of proteins orienting perpendicularly to the surface increases since a protein needs a small area to adsorb perpendicularly to the surface. The perpendicular orientation is usually energetically favorable because of the possibility for the protein to form additional bonds to the surface^37^. Moreover, hydrophobic bacteria prefer to adhere to hydrophobic surfaces, and hydrophilic bacteria adhere well to hydrophilic surfaces^38, 39^. The SRB used in our study is hydrophilic^40^. The maximum contact angle measured on the steel anodizing at 2.0 V proves the best hydrophobicity for the nanopatterned steel at this condition, which thus possesses the best property against SRB adhesion. Furthermore, it is noted that, from Supplementary Fig. 6, although the cellular morphology of SRB on the surface of anodized samples (2.0 V, 3.0 V and 4.0 V) are not as dense as that on bare steel, they are still integral. This indicates that SRB are not killed on the specimen surface. The decreased bacterial number is attributed to the anti-adhesion effect of the nanostructured anodizing film.

The one-step anodization technique reported in this work has the potential to replace the conventional methods, e.g., biocides and anti-biofouling coatings^41, 42^, for anti-adhesion of bacteria such as SRB on carbon steels. We find that, at the anodizing potential of 2.0 V, a homogeneous, compact nanostructured film is formed, which possesses the best hydrophobicity (with a water contact angel of 118.53°) and anti-adhesion performance to SRB (a 23.5 times of reduction of SRB adhesion compared to bare steel). The main components of the nanostructured film contain Fe_2_O_3_, have a good mechanical strength and chemical stability in aqueous environments^43^. Our technique to form a nanostructured film on carbon steel is simple, economic and environment-friendly, providing a promising approach to for industry-area fabrication. Furthermore, the Fe_3_O_4_ and α-Fe_2_O_3_ nanoparticles formed during anodization on carbon steel are multifunctional, such as magnetism and photocatalytic activity^44^, offering bright perspectives in applications in a wide variety of areas.

## Methods

### Material and specimen preparation for anodization

Specimens used in this work were cut from a X100 steel plate, with a chemical composition (wt%): C 0.07, Mn 1.76, Si 0.1, Ni 0.154, Cr 0.016, Mo 0.2, V 0.005, Cu 0.243, Al 0.027, S 0.005, P 0.018, and Fe balance. The specimens used for anodization were machined into rectangular shape, with a dimension of 10 mm × 10 mm × 1 mm. They were embedded into epoxy resin, leaving an exposed area of 100 mm^2^. The exposed surface was sequentially ground by emery papers up to 1,200 grit, then polished by 0.5 µm diamond paste, and degreased in ethanol using an ultrasonic bath, rinsed with deionized water, and dried by highly purified nitrogen.

The prepared steel specimen, which was used as an anode, and an X100 steel strip (cathode, with a dimension of 100 mm × 10 mm × 1 mm) were immersed into a thermostatic beaker containing 50 wt% NaOH solution, and connected to the positive and negative terminals of a direct current power supply, respectively. The experimental setup is shown in Supplementary Fig. 1a. The distance between the anode and the cathode was 50 mm. The solution was stirred by a magnetic bar at 600 rad/min during anodization. The temperature of the solution was monitored by a thermometer, and maintained at 30 °C through a water bath. The steel specimen was anodized for 10 min at various potentials. After anodization, the specimen was removed and washed with deionized water and ethanol, and dried by high-purity (99.999%) nitrogen.

Prior to anti-bacterial testing, the samples were sterilized by exposure to ultraviolet radiation for 30 min.

### Surface characterization

The morphology of the anodized steel specimens was characterized using a field emission scanning electron microscope (FEI Quanta 250 FEG). When using the SEM to observe the surface morphology of the anodized steel after 18 h of immersion in SRB medium, the specimen was washed with PBS solution for three times, immersed in 2.5% glutaraldehyde solution for 2 h, and then washed with PBS solution for three times. After that, the specimen was dehydrated with different concentrations of ethanol (30%, 50%, 70%, 90 and 100% for 15 min each), fully dried in high-purity (99.999%) nitrogen.

An atomic force microscope (Keysight 5500 scanning probe microscope system) was used for topographic characterization on the anodized steels. A scanner carrying a rectangular cantilever with a spring constant of 48 N/m (resonant frequency 150 kHz, apex radius <10 nm) was placed above the specimen. The scanning mode was configured as tapping, with a scanning rate of 0.5 Hz and a resolution of 512 × 512 pixel. The supplied software was used to create 3-dimensional topographic images and calculate the surface roughness.

The composition of the film formed on the anodized steels was characterized by Raman spectra, which were recorded through a Witec alpha 300 R Confocal Raman Microscope (WITec GmbH, Germany) using a 532 nm laser source. Integration time was 60 seconds with 3 accumulations.

Bacterial attachment on the anodized steel specimens was observed by a confocal laser scanning microscope (CLSM, Olympus FV-1000). After 18 h of immersion in the SRB-containing PBS solution, the steel specimens were washed with a sterile PBS solution, and then stained with a fluorescent dye (Molecular Probes™ FilmTracer™ LIVE/DEAD® Biofilm Viability Kit) in darkness according to the manufacturer’s procedure. The Kit utilized the mixture of SYTO™ 9 green fluorescent nucleic acid stain and red-fluorescent nucleic acid stain, i.e., propidium iodide (PI). The SYTO 9 stain could generally label all bacteria in a population, and the PI penetrated those bacteria with damaged membranes, causing a reduction in the SYTO 9 stain fluorescence while both dyes were present. Therefore, in this work, the SRB with intact cell membranes stained fluorescent green, whereas the SRB with damaged membranes stained fluorescent red. The tests were conducted in an anaerobic glove box.

Water contact angles were measured on the steel surface using a contact angle meter (100-26-TH, Ramé-hart Instrument Co.), which was combined with a video camera and software for image capture and analysis. A sessile water droplet of ∼0.75 µL was placed on the steel surface using a micro syringe. The image was captured within 5 s of the water drop placement on the specimen.

### Bacterium culturing and anti-adhering test

The SRB (*Desulfovibrio desulfuricans* subsp. *desulfuricans* (Beijerinck) Kluyver and van Niel) used in this work were purchased from American Type Culture Collection (ATCC). The following procedure was used to prepare the culture solution. Chemicals including 2.0 g MgSO_4_, 1.0 g CaSO_4_, 0.5 g K_2_HPO_4_, 0.5 g (NH_4_)_2_SO_4_, 5 g sodium citrate, 3.5 g sodium lactate and 1.0 g yeast extract were added to 1 L of deionized water. The sealed mixture was autoclaved at 121 °C for 20 min. After cooling in air to ambient temperature, the culture medium was purged with N_2_/CO_2_ (9:1) gas for 20 min to remove oxygen until dissolved oxygen is lower than 0.4 mg/L, which is measured using a dissolved oxygen meter (ExStik DO600). The pH of the prepared culture medium was adjusted to 7.5 using 1 M NaOH solution. The SRB were then added in the medium for growth at 30 °C. The bacterial growth curves in the culture medium and PBS solution were measured by the most probable number (MPN) method.

After 4 days of SRB culturing, the concentration of SRB cells is increased to 9.26 × 10^7^ cfu/mL, as seen in Supplementary Fig. 5a. The 200 mL culture medium were washed for 3 times using PSB (pH = 7.4) to eliminate the sulfide and metabolic products in the culture medium. The SRB cells were then inoculated to 200 mL PBS solution, and the concentration of SRB cells is about 9.07 × 10^7^ cfu/mL (Supplementary Fig. 5b). The steel specimens were immersed in the SRB-containing PBS solution in a 30 °C incubator in darkness for 18 h for anti-adhering testing. The testing was performed in duplicate, with each one using three parallel steel specimens which were under various anodizing treatments. All the tests were conducted in an anaerobic glove box.

### Electrochemical measurements

Electrochemical measurements were conducted on the anodized steel specimens in the anaerobic PBS solution on a three-electrode electrochemical cell, where the anodized steel specimen was used as working electrode, a carbon rod as counter electrode, and a SCE as reference electrode. The OCP of the steel specimens was monitored used an electrochemical workstation (Gamry reference 600) in a water bath of 30 °C for 18 h.

The potentiodynamic polarization curve of X100 steel in 50 wt% NaOH solution was measured at a potential scanning rate of 0.33 mV/s after the OCP of the steel reached a steasy state value.

## Electronic supplementary material

Supplementary information
