# Supplementary material for: Nanopatterning of steel by one-step anodization for anti-adhesion of bacteria
Source: Sci Rep. 2017 Jul 13;7:5326. doi: 10.1038/s41598-017-05626-0 (PMC5509660; doi:10.1038/s41598-017-05626-0)
Supplement: Supplementary file 1 — Supplementary information [file 41598_2017_5626_MOESM1_ESM.pdf]

Supplementary information

Nanopatterning of steel by one-step anodization for anti-adhesion of bacteria

Shiqiang Chen**, Yuan Li** & Y. Frank Cheng*

* Department of Mechanical & Manufacturing Engineering, University of Calgary, Calgary, Alberta, T2N 1N4, Canada. ** These authors contributed equally to this work. Correspondence and requests for materials should be addressed to Y.-F.C. (email: fcheng@ucalgary.ca).

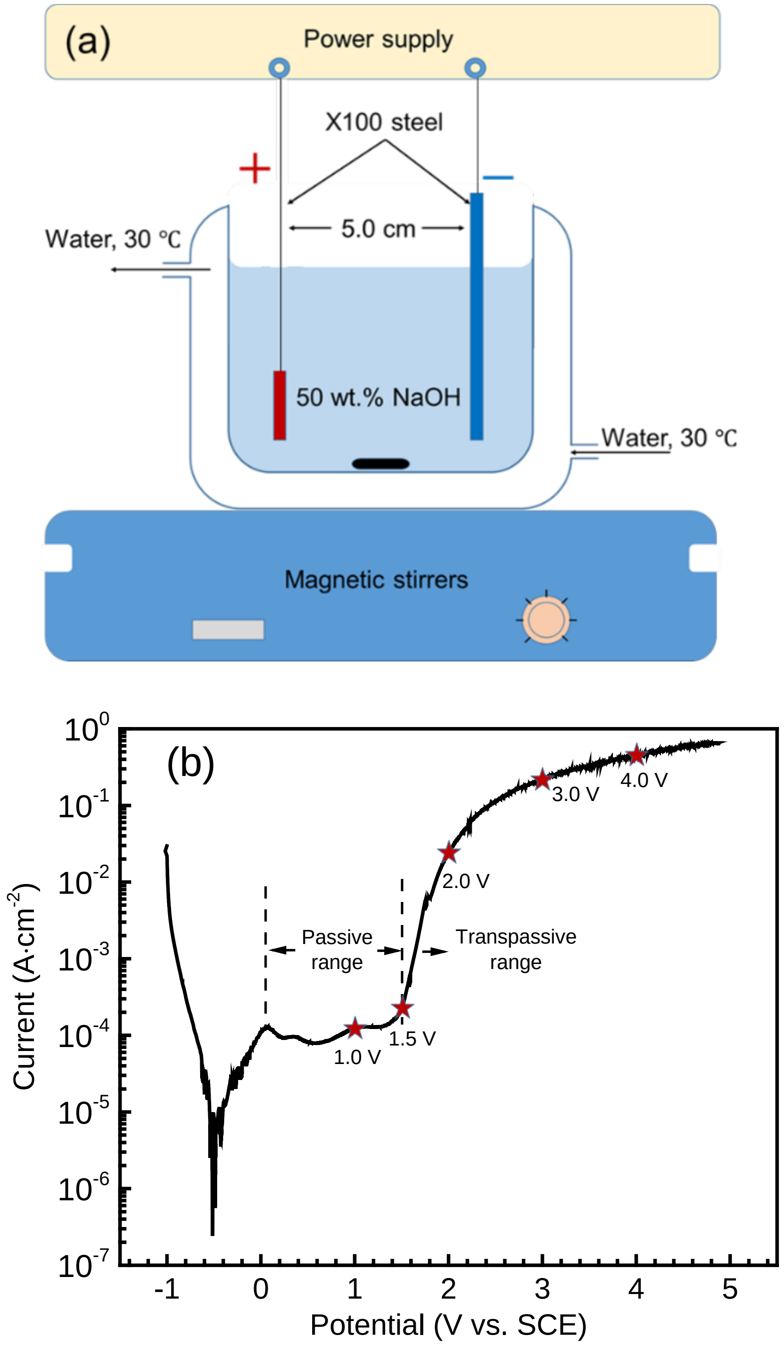

Supplementary Fig. 1 │ (**a**) Schematic setup for anodization of X100 carbon steel. (**b**) Potentiodynamic polarization curve of X100 steel in 50 wt% NaOH solution. The anodic curve includes two sections. When the potential is between 0.08 V and 1.5 V vs. SCE, the steel is in passivity. When the potential is from 1.5 to 5 V vs. SCE, the steel is in transpassive range. In this work, the potentials of 1.0 V, 1.5V, 2.0V, 3.0V and 4.0 V are selected as the anodizing potentials, as marked with red stars.

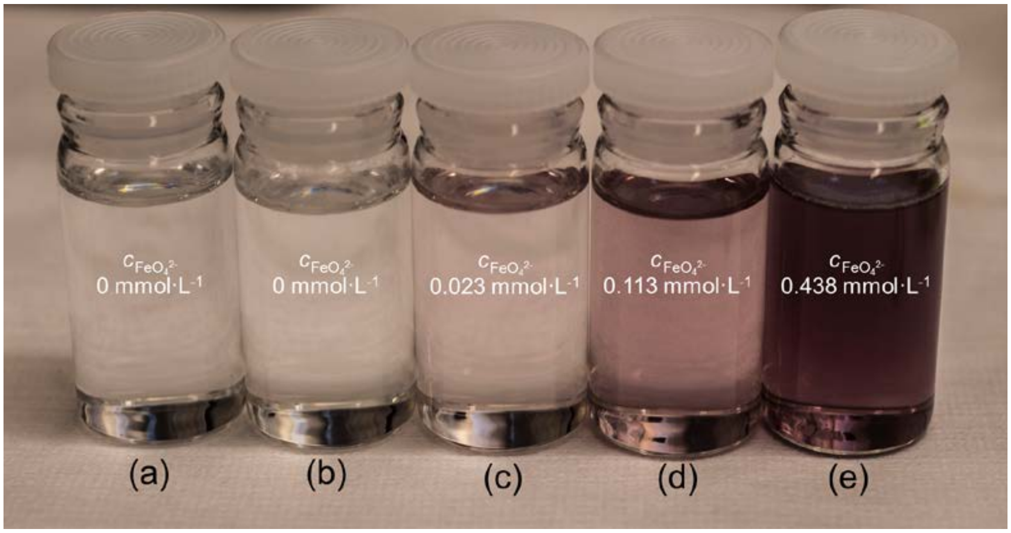

Supplementary Fig. 2 │ Digital photos of the electrolyte used for anodization at (**a**) 1.0 V, (**b**) 1.5 V, (**c**) 2.0 V, (**d**) 3.0 V and (**e**) 4.0 V. The concentrations of FeO_4^{2-} in each electrolyte are given.

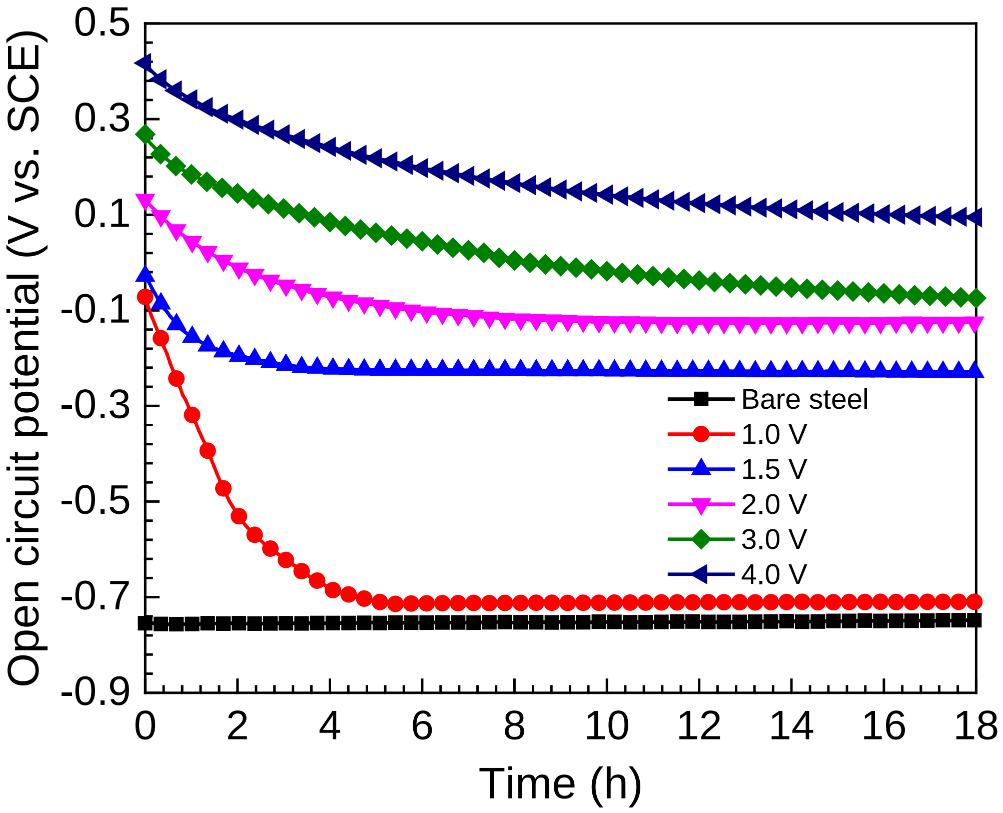

Supplementary Figure 3 | Evolution of open circuit potential of bare steel and the anodized steels at various potentials in PBS solution as a function of time. With the increasing time, the potential of bare steel keeps unchanged, while those of the anodized steels decrease and then reach steady-state values after 18 h of immersion.

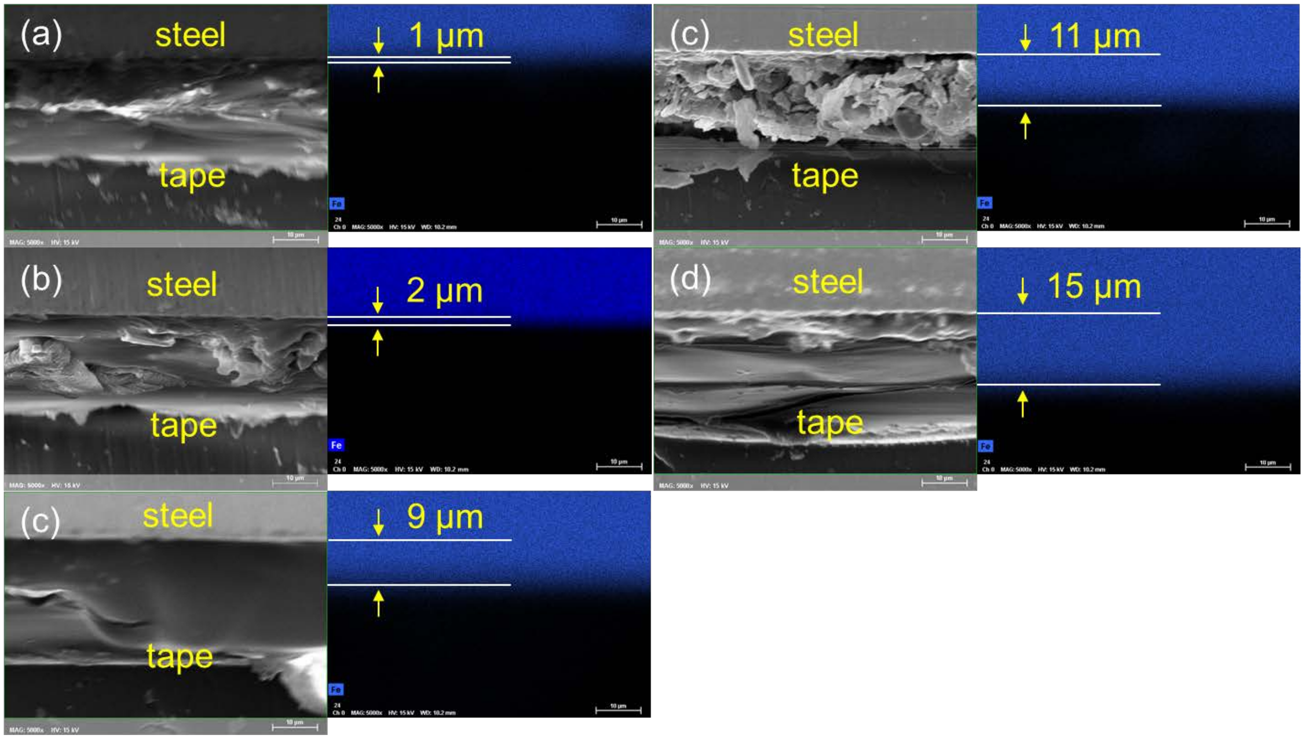

Supplementary Fig. 4 │ SEM views and Fe element maps of the cross section of anodizing films formed at potentials of (**a**) 1.0 V, (**b**) 1.5 V, (**c**) 2.0 V, (**d**) 3.0 V and (**e**) 4.0 V. The thickness of the film increases with the potential.

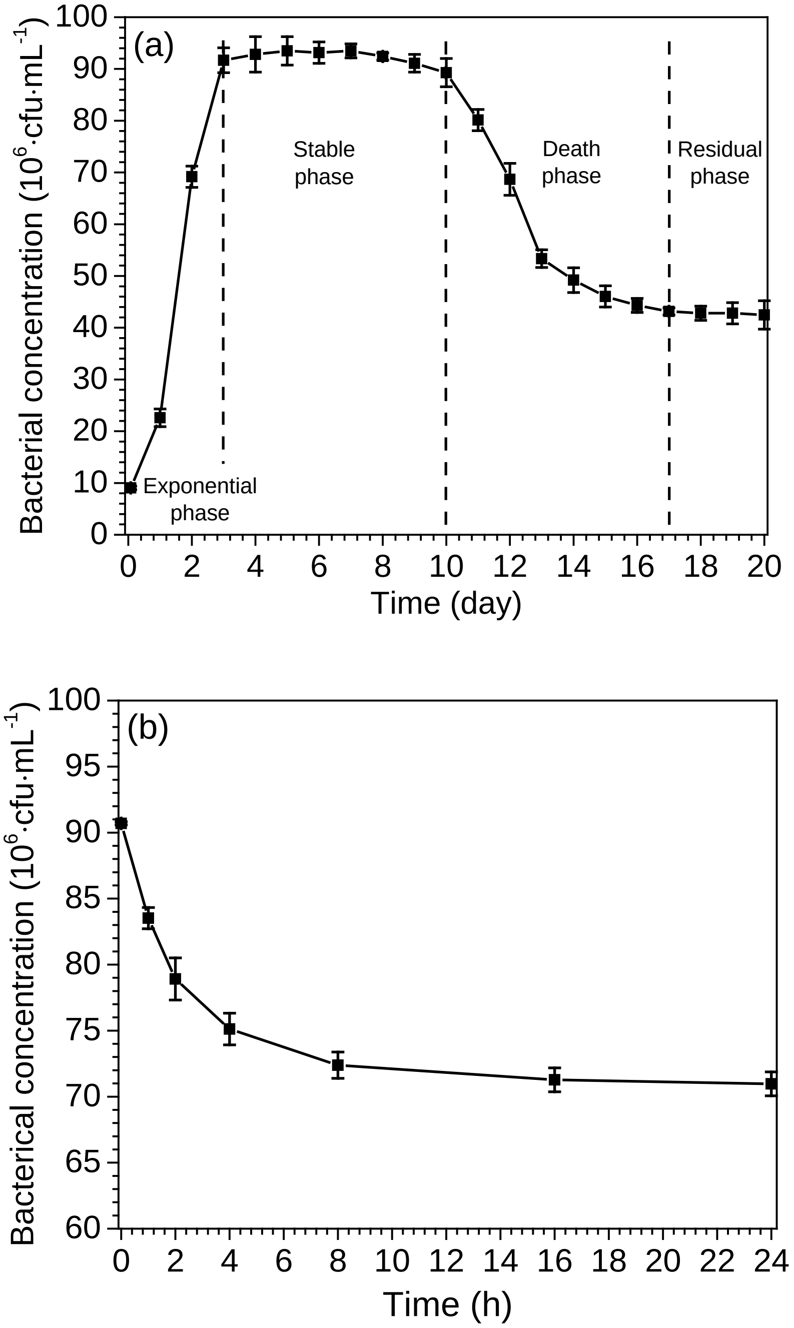

Supplementary Figure 5 │ The growth curve of SRB in (**a**) culture medium and (**b**) PBS solution. From the third day to the tenth day, the SRB growth maintains a stable metabolic activity in the culture medium, which is called stable phase. The SRB in this stage are used for anti-adhering testing. After 8 h of culturing, the bacterial concentration keeps stable around 7.1×10^7 cfu/mL, which indicates that SRB are alive and maintain metabolic activity in the oligotrophic PBS solution.

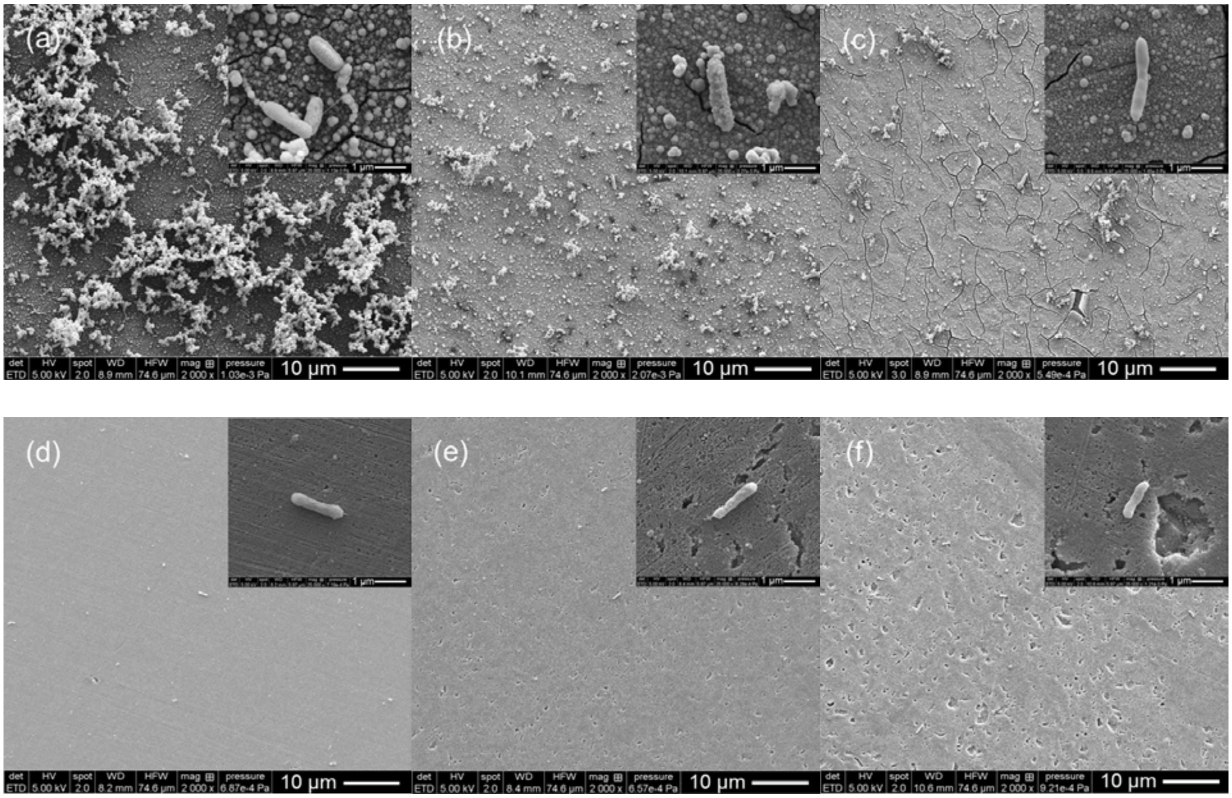

Supplementary Fig. 6 │SEM views of (**a**) bare steel, and (**b-f**) the steels anodizing at (**b**) 1.0 V, (**c**) 1.5 V, (**d**) 2.0 V, (**e**) 3.0 V and (**f**) 4.0 V after 18 h of immersion in the SRB-containing PBS solution.
